# Effect of an Internet–Delivered Cognitive Behavioral Therapy–Based Sleep Improvement App for Shift Workers at High Risk of Sleep Disorder: Single-Arm, Nonrandomized Trial

**DOI:** 10.2196/45834

**Published:** 2023-08-22

**Authors:** Asami Ito-Masui, Ryota Sakamoto, Eri Matsuo, Eiji Kawamoto, Eishi Motomura, Hisashi Tanii, Han Yu, Akane Sano, Hiroshi Imai, Motomu Shimaoka

**Affiliations:** 1 Emergency and Critical Care Center Mie University Tsu Japan; 2 Department of Medical Informatics Mie University Hospital Tsu Japan; 3 Department of Molecular Pathology & Cell Adhesion Biology Mie University Graduate School of Medicine Tsu Japan; 4 Department of Neuropsychiatry Mie University Graduate School of Medicine Tsu Japan; 5 Center for Physical & Mental Health Mie University Tsu Japan; 6 Department of Electrical & Computer Engineering Rice University Houston, TX United States

**Keywords:** shift worker sleep disorder, internet-based cognitive behavioral therapy, mobile apps, fitness tracker, subjective well-being, machine learning, mobile phone

## Abstract

**Background:**

Shift workers are at high risk of developing sleep disorders such as shift worker sleep disorder or chronic insomnia. Cognitive behavioral therapy (CBT) is the first-line treatment for insomnia, and emerging evidence shows that internet-based CBT is highly effective with additional features such as continuous tracking and personalization. However, there are limited studies on internet-based CBT for shift workers with sleep disorders.

**Objective:**

This study aimed to evaluate the impact of a 4-week, physician-assisted, internet-delivered CBT program incorporating machine learning–based well-being prediction on the sleep duration of shift workers at high risk of sleep disorders. We evaluated these outcomes using an internet-delivered CBT app and fitness trackers in the intensive care unit.

**Methods:**

A convenience sample of 61 shift workers (mean age 32.9, SD 8.3 years) from the intensive care unit or emergency department participated in the study. Eligible participants were on a 3-shift schedule and had a Pittsburgh Sleep Quality Index score ≥5. The study comprised a 1-week baseline period, followed by a 4-week intervention period. Before the study, the participants completed questionnaires regarding the subjective evaluation of sleep, burnout syndrome, and mental health. Participants were asked to wear a commercial fitness tracker to track their daily activities, heart rate, and sleep for 5 weeks. The internet-delivered CBT program included *well-being prediction*, *activity and sleep chart*, and *sleep advice*. A job-based multitask and multilabel convolutional neural network–based model was used for well-being prediction. Participant-specific sleep advice was provided by sleep physicians based on daily surveys and fitness tracker data. The primary end point of this study was sleep duration. For continuous measurements (sleep duration, steps, etc), the mean baseline and week-4 intervention data were compared. The 2-tailed paired *t* test or Wilcoxon signed rank test was performed depending on the distribution of the data.

**Results:**

In the fourth week of intervention, the mean daily sleep duration for 7 days (6.06, SD 1.30 hours) showed a statistically significant increase compared with the baseline (5.54, SD 1.36 hours; *P*=.02). Subjective sleep quality, as measured by the Pittsburgh Sleep Quality Index, also showed statistically significant improvement from baseline (9.10) to after the intervention (7.84; *P*=.001). However, no significant improvement was found in the subjective well-being scores (all *P*>.05). Feature importance analysis for all 45 variables in the prediction model showed that sleep duration had the highest importance.

**Conclusions:**

The physician-assisted internet-delivered CBT program targeting shift workers with a high risk of sleep disorders showed a statistically significant increase in sleep duration as measured by wearable sensors along with subjective sleep quality. This study shows that sleep improvement programs using an app and wearable sensors are feasible and may play an important role in preventing shift work–related sleep disorders.

**International Registered Report Identifier (IRRID):**

RR2-10.2196/24799.

## Introduction

### Interventions for Shift Work–Related Sleep Disorder

Shift work is common among the working population, with approximately 20% of individuals engaged in shift work [1]. However, shift workers are at a heightened risk of developing sleep disorders, with studies showing that workers on consecutive night shifts or fixed night shifts are more prone to sleep deprivation [2,3]. Shift worker sleep disorder (SWSD) is a circadian rhythm disorder that presents as excessive sleepiness or insomnia associated with shift work. Although the prevalence of SWSD varies among different definitions, studies show that 10% to 20% of shift workers present SWSD features [4,5]. SWSD is distinguished from chronic insomnia in that SWSD primarily results from misalignment of the circadian rhythm because of shift work. However, it is difficult to make this distinction as SWSD and chronic insomnia may present overlapping features. In a study, approximately half (40.9%) of hospital shift workers screened as positive for sleep disorders, with positive screening associated with an 83% increased incidence of adverse safety outcomes [6]. In recent years, multimodal longitudinal studies using wearable sensors have provided more detailed insight into the sleep disturbances of shift workers. Using physiological time series collected from a commercial fitness tracker, Fitbit (Fitbit Inc), regular routine patterns such as sleep were reflected through heart rate time series data [7,8]. The data also showed that night shift nurses were more sedentary and reported lower sleep quality [9].

Cognitive behavioral therapy (CBT) is a treatment aimed at restructuring undesired thinking and modifying behavioral patterns through self-reflection and interactions with physicians. There are 4 fundamental psychotherapy techniques used in CBT: cognitive restructuring, behavioral activation, exposure, and problem-solving [10,11]. CBT is recommended as the first-line treatment for insomnia, with a meta-analysis showing a remarkable improvement in sleep outcomes [12]. Applying the same principles, internet-delivered CBT programs for insomnia are rapidly increasing, with outstanding results. A systematic review of randomized controlled trials found that sleep efficiency, insomnia severity index, and total sleep time improved with internet-delivered behavioral therapy for insomnia in adults [13]. Most studies included physician or therapist involvement in the intervention, mostly to provide weekly feedback based on sleep diaries and questionnaires provided by the participants [14-16].

Several studies have adapted CBT for insomnia in shift workers. Some components of CBT for insomnia, such as sleep hygiene promotion and relaxation techniques, are considered to be equally effective for SWSD. In addition, similar to insomnia, sleep diaries or actigraphs are recommended to assess sleep disturbances and to evaluate the effects of treatment. However, the literature suggests that simply applying CBT for insomnia in shift workers may not result in an improvement in insomnia symptoms. In a study, CBT for insomnia was effective when shift workers were excluded from the analysis [17]. Another study found that among shift workers diagnosed with insomnia, those with SWSD features showed less prominent improvement [18]. The authors concluded that as SWSD stems from misalignment of the circadian rhythm, psychoeducational treatment has little effect. However, other studies suggest that adapting CBT for insomnia specifically for shift workers is effective. For example, instead of sleep restriction, shift workers may benefit from other strategies such as structured naps and anchor sleep. Other strategies specific to shift workers include appropriate caffeine intake and light exposure. Booker et al [19] conducted a study to investigate the impact of an individual shift work management coaching program on various factors such as sleep hygiene, insomnia symptoms, and depression symptoms. The program included interventions such as education on sleep hygiene, implementing scheduled naps and sleep periods, and managing light exposure during work hours. The results revealed a significant improvement in participants’ sleep hygiene, reduction in insomnia symptoms, and alleviation of depression symptoms. Lee et al [20] provided home-based sleep enhancement training for shift work nurses, resulting in improvements in sleep quality. Peter et al [21] compared web-based CBT and face-to-face outpatient treatment for shift workers and found a significant improvement in sleep efficacy in both groups. The CBT elements for the web-based groups included sleep restriction, sleep hygiene education, and relaxation techniques and were provided by semistandardized emails [21]. Together, these studies indicate that modified CBT for insomnia, which consists of combined strategies personalized to each participant and guided by physicians, are effective in improving sleep and well-being for shift workers.

### Shift Workers’ Well-Being and Well-Being Prediction

Several studies have shown a correlation between poor sleep patterns resulting from shift work and an increased risk of burnout among various professional groups, including police officers [22], physicians working in the intensive care unit (ICU) [23], nurses, and other shift work occupations [3]. Research has shown that improving sleep has a positive impact on mental well-being. Several studies examining CBT for insomnia have shown improvements in mental health and well-being and sleep parameters [13,24,25]. This has also been demonstrated in studies aiming to improve sleep in shift workers [19,21]. Taken together, these studies support the notion that improving sleep has a high probability of enhancing the well-being of shift workers. However, most studies that assessed mental health and well-being among shift workers often relied on pre- and postintervention questionnaires, providing limited insights into continuous changes over time. In this study, we collected daily well-being scores from the participants, allowing us to capture the fluctuating nature of well-being, similar to sleep patterns. Our primary aim was to evaluate the changes in well-being scores using daily data, recognizing that well-being can vary on a day-to-day basis.

The second aim was to provide “well-being prediction” feedback to the participants. Previous studies have shown that self-monitoring prompts users to self-reflect and improve their behaviors, such as eating and exercise. Reflection is an essential activity in personal informatics apps that enable users to generate insight for self-improvement and make behavioral or mental changes [26]. Tracking feelings and daily activities leads to improved self-care and emotion regulation through better self-awareness and understanding of the correlations between their activities and feelings [27]. Some personal informatic apps simply collect and present data, relying on the user to analyze the effect of past behavior and the present state, whereas other apps use algorithms that explain these effects. Algorithms that predict the future based on daily activity and habits aim to provide users with a tool that supports self-reflection and promotes behavioral change based on these insights. For example, Kim et al [28] reported the effect of an algorithm-assisted stress management system that determines stress levels along with explanations based on everyday activities. Hollis et al [29] showed that forecasting future moods based on past mood data and trigger activities improved mood and emotional self-awareness compared with controls who simply monitored their past. DeMasi et al [30] used models predicting well-being from activity and sleep measures from smartphones, demonstrating the feasibility and capability of automatic mood tracking. Nosakhare and Picard [31] used a well-being prediction model using data from wearable sensors and surveys with the intention to recommend healthy behaviors that will improve the state of well-being. Therefore, in this study, we implemented well-being prediction to promote self-reflection, which is an essential element of CBT.

### Objectives

This study aimed to evaluate the effect of a physician-assisted, internet-delivered CBT intervention with machine learning–based well-being prediction on sleep duration and well-being of shift workers at high risk of sleep disorders. The study followed a single-arm prospective design, with a baseline period of 1 week, followed by a 4-week intervention period. The participants were shift workers from the ICUs of 2 hospitals. Sleep duration and other relevant outcomes were assessed using a combination of an internet-delivered CBT app and a fitness tracker.

## Methods

### Study Design and Settings

This prospective interventional study aimed to evaluate the effect of internet-delivered CBT on shift workers with a high risk of sleep disorders. This study involved shift workers, including physicians and nurses, employed in 2 ICUs in Japan. Participants were instructed to wear a wrist-worn fitness tracker 24 hours a day and answer daily questionnaires about their well-being and activities. Internet-delivered CBT was provided through an app installed on the participants’ phones. We compared the total sleep duration, subjective well-being, and other variables in the baseline and intervention periods.

The participants were recruited using flyers and email, both including the same content that stated the aim of the research project, the participant’s eligibility requirements, intervention methods, and participant’s rights. The emails were distributed by the head of the department to all employees. The flyers were handed out at staff meetings. After the flyers and emails were distributed, a research assistant asked all eligible employees about their intention to participate. After inclusion, the participants were completely anonymous to the study team and sleep physicians who reviewed the participant’s data and provided sleep advice (see details in the *Intervention* section). However, they were in the same working environment as the researchers in the study team. To maintain anonymity, each participant was allocated a study ID, and the researchers in the study team were not directly able to identify the participants after the intervention had started, although the study team (a research assistant who is not a health care provider) was able to contact the participants through email when technical issues occurred. Financial incentives were provided upon the completion of the study.

### Inclusion and Exclusion Criteria

Inclusion criteria were as follows: shift workers engaged in emergency and intensive care areas, working on a 3-shift schedule with 8-hour shifts, and a Pittsburgh Sleep Quality Index (PSQI) score ≥5. Exclusion criteria were as follows: diagnosis of sleep disorders such as sleep apnea, restless leg syndrome, and narcolepsy; previous history of psychiatric disorders; and pregnancy. Participants with these diagnoses were excluded from the study so that the intervention or sleep advice would not interfere with the present medical treatment. The participants were assessed for eligibility according to self-reported past medical history or pregnancy and the PSQI test. The participants were asked if any of the exclusion criteria applied to them.

### Study Measures and Outcomes

#### Objective and Subjective Sleep Measurements

The primary outcome of this study was to evaluate the effect of the intervention on total sleep time. In this study, we used a consumer fitness tracker called Fitbit Charge 3 (Fitbit). It is widely used in sleep research under normal living conditions because of its easy obtainability, cost efficiency, and comparability with polysomnography [32]. Primary data were obtained using the sensor’s built-in accelerometer, altitude sensor, and heart rate sensor. This wearable sensor can continuously collect the following biometric information: sleep-related information (start and end time of sleep, minutes awake during sleep, and time of each sleep stage), heart rate, and information on activity (number of steps, calories burned, and intensity of exercise). In a systematic review, the authors found that Fitbit’s sleep-staging algorithms showed no significant difference in wake-after-sleep onset and total sleep time when compared with polysomnography [33]. They concluded that although Fitbit cannot be used as a substitute for polysomnography, recent Fitbit sleep algorithm can differentiate wake from sleep better than earlier reports on actigraphy performance [33]. However, consumer fitness trackers perform poorly in detecting naps [32]. In light of these validation studies and considering the short, fragmented sleep of shift workers, we evaluated only sleep duration as a primary objective sleep outcome. For other subjective sleep measurements, we used the PSQI to evaluate subjective sleep quality. The PSQI is a self-assessed measurement of subjective sleep, consisting of 19 items and divided into 7 subscores. The total scores range from 0 to 21, with higher scores indicating poor sleep quality. A global PSQI score >5 indicates poor sleep [34]. However, studies in Japan often use a cutoff score of 5.5 [35], and several other studies including the general population often use the criteria of PSQI ≥5 to differentiate between poor and good sleepers. In this study, we used a threshold of 5 to include mild symptoms of sleep disturbance in the general working population. Participants also evaluated subjective sleep quality through the morning survey, which consisted of 5 questions (eg, “How deep did you sleep?” and “How soon did you sleep?”) rated on a scale of 1 to 5.

#### Subjective Well-Being and Mental Health State

As a secondary outcome, we evaluated the effect of the intervention on subjective well-being. Participants scored their subjective well-being using 5 categories: alertness, happiness, energy, physical health, and calmness. Participants were asked to rate their subjective well-being on a scale of 0 to 100 as part of their morning and evening daily surveys. Other questions in the daily survey included activities and habits (eg, alcohol and caffeine intake). The 5 categories of well-being and other surveys were used in a prior study of well-being prediction [36]. In addition, as an objective measurement of alertness, participants took the 3-minute psychomotor vigilance test (PVT) along with daily surveys [37]. The PVT measures the reaction time to stimuli occurring at random intervals, where an elongated reaction time indicates deterioration of cognitive performance. The participants answered the morning and evening daily surveys along with performing the 3-minute PVT on their app.

**Figure 1:**
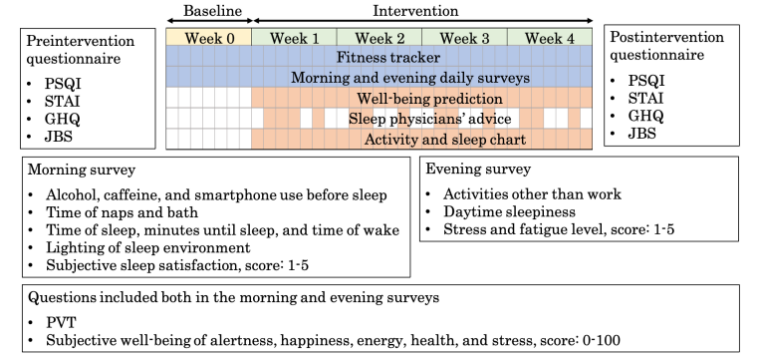
Intervention and measurement schedules. Well-being prediction and the activity and sleep chart was available to the participants every day, whereas sleep advice was provided 3 to 4 times a week. The participants answered the pre and postintervention questionnaires and daily morning and evening surveys. GHQ: General Health Questionnaire; JBS: Japan Burnout Score; PSQI: Pittsburgh Sleep Quality Index; PVT: psychomotor vigilance test; STAI: State-Trait Anxiety Inventory.

We also compared several validated questionnaires on mental health and burnout symptoms before and after the intervention (Figure 1). The 12-item General Health Questionnaire (GHQ) is a widely used screening tool to measure symptoms of psychological distress in the general population, both in research and clinical practice [38,39]. There are mainly 4 methods of scoring the GHQ-12: standard GHQ scoring, Likert scoring, modified Likert scoring, and chronic GHQ scoring. We used the standard GHQ scoring method, a common method used in research, where items are rated from a score of 0 to 1, with a total score ranging from 0 to 12 and higher scores indicating higher levels of psychological distress [40]. For the assessment of the risk of mental health disorders, a cutoff score of 4 was used [41]. We also used the Japan Burnout Score, a scale developed to assess burnout for the Japanese industry. It is based on the Maslach Burnout Inventory and is compiled into 17 items [42]. Japan Burnout Score, similar to Maslach Burnout Inventory, consists of 3 subscales: emotional exhaustion, depersonalization, and decreased personal accomplishment. Each item is scored from 1 to 5 and divided by the number of items in each subscale, with higher scores indicating higher burnout levels. Finally, the State-Trait Anxiety Inventory (STAI) evaluates an individual’s feelings of anxiety. It was developed to provide an easy and reliable scale for assessing state and trait anxiety. The STAI test has 2 subscales: state and trait. Each subscale has 20 items with a score of 1 to 4, with total scores of each subscale ranging from 20 to 80 and higher scores representing higher levels of anxiety [43]. Permission to use the STAI test was obtained from the copyright holder, Sankyobo, Kyoto, Japan.

### Sample Size and Power Calculation

Before this study, we collected data from 16 shift workers in a pilot study. The mean sleep duration was 334.68 (SD 135.1) minutes. On the basis of the pilot study data with a small sample, the mean sleep duration at week 4 of the intervention was assumed to be 30 minutes longer, with no change in SD. Assuming a 2-sided level of significance of 5%, power of 80%, and correlation coefficient of 0.8, using the SAS system (SAS Institute Inc), 66 participants were required for a significant difference to be found in the 2-tailed paired *t* test. We set the required number of participants to 70 because the dropout rate of the study was assumed to be approximately 5% of the enrolled individuals. The dropout rates of face-to-face CBT for insomnia in randomized controlled trials ranged from 0% to 8% [44]. In prior studies, the mean attrition rate of internet-based psychological intervention programs performed in the workplace was 23%, with a range of 3% to 54% [45]. We estimated that a low dropout rate could be achieved owing to the high frequency of feedback from sleep physicians.

### Intervention

**Figure 2:**
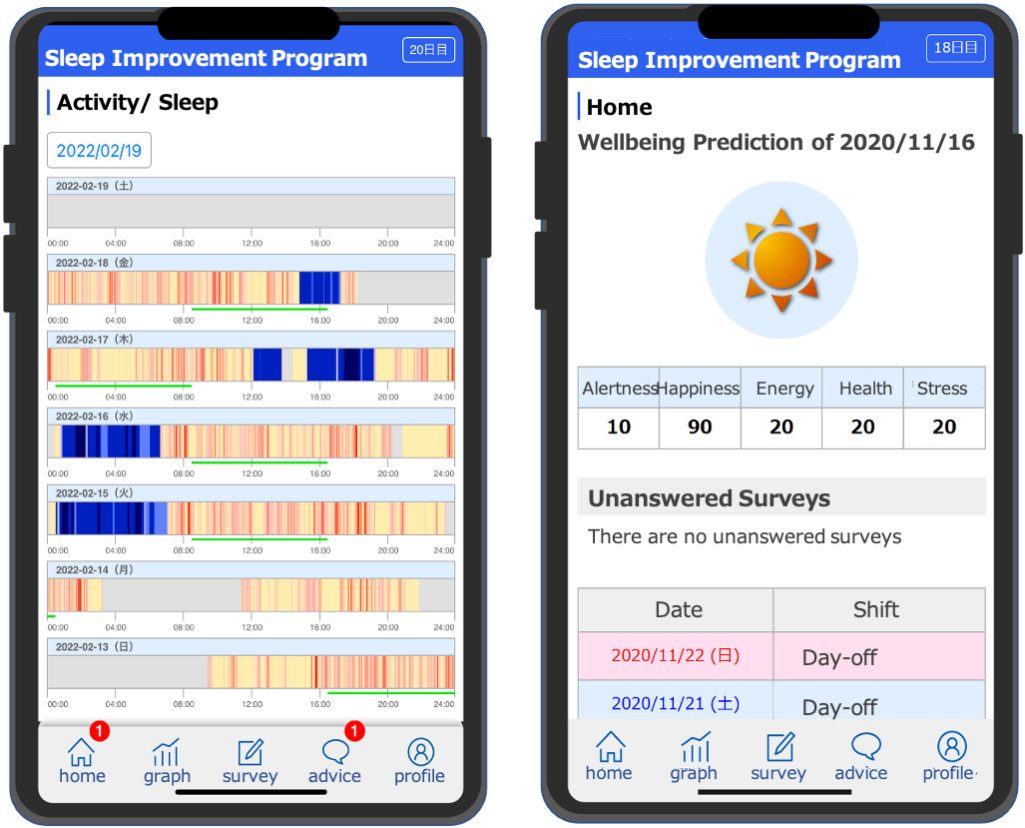
The user interface of the app, translated from Japanese to English. Left: Awake times are displayed as red bars, where higher activity levels (higher number of steps/min) are shown in deeper shades of red. Sleep times are displayed as blue bars, where deeper sleep stages are shown in deeper shades of blue. Right: The happiness score is illustrated by a graphic that resembles a weather forecast: cloudy for scores 0 to 50, cloudy and partly sunny for scores 60 to 80, and sunny for scores 90 to 100.

The smartphone app for the intervention was developed and made available as a Progressive Web Application so that it could be used on an iOS or Android device (Figure 2). The smartphone app is a client of the internet-delivered CBT system and is responsible for collecting manual entry information from users, such as daily surveys. The system also provides research coordinators and sleep physicians with a management portal for writing and sending advice to users. The system can further run the preprogrammed well-being prediction model using the daily data collected from the fitness tracker. It was then fed back to the user’s app. The 3 main components of the program were well-being prediction, sleep advice, and data visualization.

Well-being prediction is an essential component of an app. On the basis of earlier studies on machine learning and subjective well-being [36], we categorized well-being into 5 components: alertness, happiness, energy, physical health, and calmness. Well-being labels were predicted using wearable sensors and daily surveys. Well-being prediction was based on these 5 self-reported labels that were collected twice daily (8 AM and 8 PM) and reported on a scale of 0 to 100. To predict well-being scores, we developed a job-based multitask and multilabel convolutional neural network–based well-being prediction model using pilot data from 31 participants with 906 days of data collection [46]. A total of 23 daily features were extracted from the fitness tracker and daily surveys. These included biobehavioral features such as sleep duration, sleep variability, number of steps, mean and variability of heart rate for the previous 7 days, work-related features such as shift schedule and working hours, and daily habits such as alcohol and caffeine intake. The model extracted high-level features using convolutional kernels and simultaneously predicted 5 well-being scores for physicians and nurses. The prediction performance of the proposed model was compared with that of the control models, including job role–based multitask only and multilabel only (physicians only, nurses only, and all participants), and baseline models, such as support vector machine and random forest. The proposed model achieved the best performance in almost all evaluations performed.

The second essential component is personalized sleep advice from physicians. Participants received personalized sleep advice from 2 board-certified psychiatrists specializing in sleep disorders and CBT 3-4 times a week. The advice was suggested after the psychiatrists reviewed the fitness tracker data and surveys, without seeing the well-being prediction results. This ensured that sleep advice was solely based on the physician’s clinical judgment. Physicians chose 3 to 5 messages among 23 fixed-format sleep advice messages (eg, “Avoid alcohol before you go to sleep” and “Try to wake up at a consistent time.”; Multimedia Appendix 1). The participants were asked to send their feedback concerning the advice using a thumbs-up or thumbs-down icon. They were also able to send feedback messages to physicians. However, to ensure equal investment of effort among the participants, the physicians did not respond to the comments. This feedback feature from the participants to the physicians was designed to ensure that the participants read the advice in a timely fashion and to promote interactive participation, thereby potentially enhancing their motivation to continue.

On the “activity and sleep chart” tab, the participants were readily able to see how much activity and sleep they had in 24 hours for the past 7 days, along with their shift schedule. These data were retrieved every hour from the Fitbit server using its web application programming interface. The intervention has been described in detail in a previous study protocol [47].

### Statistical Analyses

Statistical analyses were performed using SPSS Statistics for Macintosh (version 25.0; IBM Corp). The baseline characteristics of the study population were expressed as mean and SD for quantitative variables. For the primary outcome of this study, the mean sleep duration at week 4 of the intervention and the means and SDs of sleep duration were calculated at both the baseline data collection period and at week 4 of the intervention period. For the primary end point, a 2-sided paired *t* test or Wilcoxon signed rank test was performed, with a significance level of 5%. The secondary outcomes of this study were activity (calories burned and steps taken), subjective well-being, reaction time, and subjective quality of sleep. The median for baseline (week 0) and week 4 of the intervention were calculated, and a 2-tailed paired *t* test or Wilcoxon signed rank test was performed at a 2-sided significance level of 5%. The pre and postintervention PSQI, GHQ, and STAI scores were similarly evaluated.

### Ethics Approval

This study was approved by the Clinical Research Ethics Review Committee of Mie University Hospital, Tsu, Japan (H2020-083). This study was approved by the ethics committee of the Suzuka General Hospital (review number 254). All procedures were performed in accordance with the ethical standards of the Institutional Research Committee and 2013 Declaration of Helsinki. This study was registered in the University Hospital Medical Information Network Clinical Trials Registry on May 1, 2020 (ID: UMIN000040547).

## Results

### Baseline Characteristics of the Participants

A total of 62 shift workers in the ICU or emergency department were included, and 1 participant was excluded owing to insufficient data. Of the 61 remaining participants, their age ranged from 21 to 55 (mean 32.9, SD 8.3) years. Most of the participants were nurses (57/61, 93%) and female (50/61, 82%). Data were collected from 2 hospitals: a large-scale university hospital (46/61, 75%) and a municipal hospital (15/61, 25%; Table 1). Recruitment ended before the inclusion of the intended number of participants because the working arrangements in 1 of the ICUs changed from a 3-shift schedule to a 2-shift schedule during the study period.

According to the inclusion criteria, all participants had some degree of sleep disorder as defined by a PSQI score ≥5. The mean baseline PSQI score was 9.10. The study population showed high burnout scores for all 3 components (emotional exhaustion, depersonalization, and personal achievement). According to a study targeting nurses in Japan [48], the mean burnout scores for emotional exhaustion, depersonalization, and personal achievement in groups of nurses who wanted to leave the hospital were 3.27, 2.06, and 3.51, respectively. The mean GHQ score was 7.45, which was higher than the scores for nurses in the general ward in a reported study, with a mean of 5.42 (SD 3.29) [49]. A high risk for mental disorders was indicated in 51% (31/61) of the participants using a cutoff score of 4 with the standard GHQ scoring method [41]. State and trait anxiety was present in 64% (39/61) and 79% (48/61) of the participants, respectively, using a cutoff score of 42.

**Table 1:** Demographic characteristics of the participants (n=61).

Demographic characteristics			Value
**Age (years)**			
	Mean (SD)	32.9 (8.37)	
	20-29, n (%)	29 (48)	
	30-39, n (%)	19 (31)	
	40-49, n (%)	10 (16)	
	50-59, n (%)	3 (5)	
**Sex, n (%)**			
	Female	50 (82)	
	Male	11 (18)	
**Occupation, n (%)**			
	Nurse	57 (93)	
	Physician	4 (7)	
**Hospital description, n (%)**			
	University hospital	46 (75)	
	Municipal hospital	15 (25)	
**Chronotype, n (%)**			
	Definitely morning type	0 (0)	
	Moderately morning type	9 (15)	
	Intermediate	47 (77)	
	Moderately evening type	3 (5)	
	Definitely evening type	2 (3)	
Routine use of sleep medication, n (%)			1 (2)
PSQI^a^ (score), mean (SD)			8.74 (2.72)
**GHQ^b^ (score), mean (SD)**			7.45 (3.31)
	Indication of mental illness (≥4)	31 (50.8)	
JBS^c^–emotional exhaustion, mean (SD)			3.57 (0.86)
JBS-depersonalization, mean (SD)			2.17 (0.77)
JBS–personal achievement, mean (SD)			3.77 (0.77)
**STAI^d^ (state), mean (SD)**			45.96 (10.6)
	Indication of state anxiety (≥42)	39 (63.9)	
**STAI (trait), mean (SD)**			49.9 (9.81)
	Indication of trait anxiety (≥42)	48 (78.6)	

^a^PSQI: Pittsburgh Sleep Quality Index.

^b^GHQ: General Health Questionnaire.

^c^JBS: Japan Burnout Score.

^d^STAI: State-Trait Anxiety Inventory.

### Changes in Sleep Duration and Other Sleep-Related Variables

**Figure 3:**
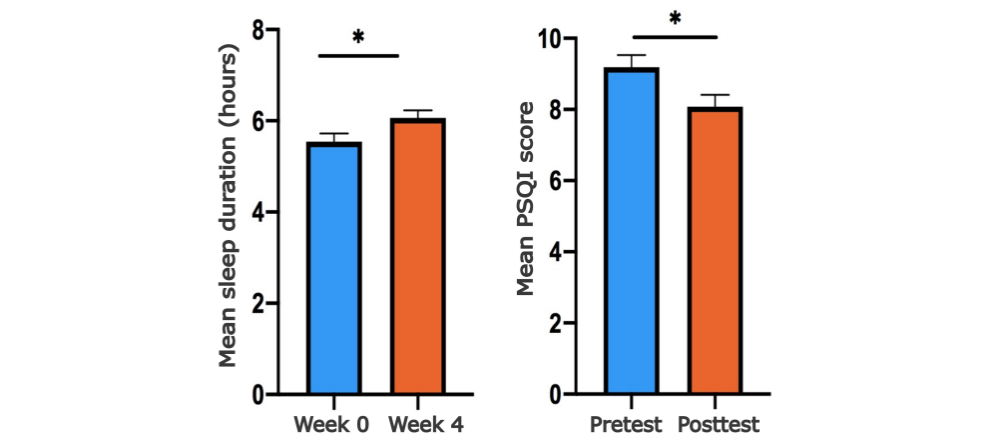
The mean sleep duration and the Pittsburgh Sleep Quality Index (PSQI) before and after the intervention. Mean sleep duration improved from 5.54 hours to 6.06 hours (*P*=.02). The PSQI improved from 8.74 to 7.84 (*P*=.003). *P<.05.

At baseline, the mean daily sleep duration over 7 days was 5.54 hours. In the fourth week of the intervention, a statistically significant increase in daily sleep duration was observed, with a mean duration of 6.06 hours (*P*=.02; Table 2). Subjective sleep quality as measured by the PSQI also showed statistically significant improvement from baseline after the intervention (9.10 vs 7.84; *P*=.001; Hedges *g*=−0.34; Figure 3; Table 3). According to a systematic review and meta-analysis, the clinical significance threshold for total sleep time, as measured by actigraphy, was 15 minutes, and improvement in PSQI had an effect size of 0.5 based on Hedges *g* [50]. As this study shows that sleep duration increased by 0.52 hours after intervention, but the effect size for PSQI change was <0.5, from the literature it can be considered that only improvement in sleep duration was clinically significant. A 3-way ANOVA was conducted to examine the effect of sex and age on sleep hours. There was no statistically significant interaction between the effect of sex (*F*_2,115_=0.349; *P*=.56) and age (*F*_2,115_=1.075; *P*=.35) on sleep hours, whereas the effect of the intervention showed statistical significance (*F*_1,115_=4.554; *P*=.05). The effect of sex and age was evaluated in the same manner for subjective well-being. Age had a statistically significant effect on alertness in the morning (*P*=.01), while both sex (*P*=.007) and age (*P*=.002) had a statistically significant effect on alertness in the evening (Multimedia Appendix 2).

**Table 2:** Comparison of biological data, well-being scores, subjective sleep parameters, and daily habits before and after the intervention.

Parameters			Week 0, mean (SD)		Week 4, mean (SD)		*P* value		Statistical test type
**Biological data**									
	Total sleep duration (h)	5.54 (1.36)		6.06 (1.30)		.02		Wilcoxon signed rank test	
	Steps	7678 (2922)		7471 (3058)		.26		Wilcoxon signed rank test	
	Heart rate (beats/min)	74.9 (7.75)		74.79 (6.62)		.95		2-tailed paired *t* test	
**Working hours**									
	Working hours (h)	5.67 (1.77)		5.50 (1.68)		.55		Wilcoxon signed rank test	
**Well-being**									
	Alertness (morning)	39.7 (14.0)		41.1 (14.9)		.54		2-tailed paired *t* test	
	Happiness (morning)	51.4 (11.5)		52.4 (12.3)		.35		Wilcoxon signed rank test	
	Energy (morning)	40.3 (12.9)		45.6 (15.4)		.01		2-tailed paired *t* test	
	Health (morning)	50.7 (14.1)		51.4 (13.8)		.43		Wilcoxon signed rank test	
	Relax (morning)	55.4 (13.2)		54.6 (13.9)		.80		Wilcoxon signed rank test	
	Alertness (evening)	41.0 (13.9)		41.1 (14.2)		.98		2-tailed paired *t* test	
	Happiness (evening)	55.0 (12.4)		52.9 (12.4)		.22		Wilcoxon signed rank test	
	Energy (evening)	44.7 (15.2)		46.3 (16.0)		.38		2-tailed paired *t* test	
	Health (evening)	52.4 (15.0)		52.8 (14.4)		.88		Wilcoxon signed rank test	
	Relax (evening)	57.7 (15.4)		56.6 (14.2)		.35		Wilcoxon signed rank test	
**Subjective sleep parameters**									
	Sleep depth (0-5)	3.01 (0.59)		3.16 (0.77)		.13		2-tailed paired *t* test	
	Rapid onset of sleep (0-5)	3.47 (0.73)		3.51 (0.74)		.70		2-tailed paired *t* test	
	Recovery of fatigue (0-5)	2.68 (0.60)		2.81 (0.76)		.19		2-tailed paired *t* test	
	Continuity of sleep (0-5)	3.15 (0.98)		3.04 (0.87)		.29		2-tailed paired *t* test	
	Sleep satisfaction (0-5)	2.65 (0.54)		2.84 (0.70)		.04		2-tailed paired *t* test	
**Daily habits**									
	Alcohol (cups)	0.29 (0.40)		0.27 (0.40)		.68		Wilcoxon signed rank test	
	Caffeine (cups)	0.86 (0.81)		0.73 (0.70)		.01		Wilcoxon signed rank test	
	Smartphone time before sleep (min)	7.76 (7.83)		8.77 (8.94)		.17		Wilcoxon signed rank test	
	Brightness (0-100)	21.1 (17.9)		16.1 (15.4)		.03		Wilcoxon signed rank test	
	Fatigue (0-5)	2.51 (0.58)		2.48 (0.62)		.71		2-tailed paired *t* test	
	PVT^a^ (morning)	373.7 (123.6)		397.3 (141.2)		.01		Wilcoxon signed rank test	
	PVT (evening)	375.7 (96.8)		406.6 (130.1)		.001		Wilcoxon signed rank test	

^a^PVT: psychomotor vigilance test.

**Table 3:** Comparison of questionnaires before and after the intervention.

Parameters	Before intervention, mean (SD)	After intervention, mean (SD)	*P* value	Statistical test type
PSQI^a^	9.10 (4.1)	7.84 (2.46)	.01	2-tailed paired *t* test
GHQ^b^	7.45 (3.3)	6.76 (3.0)	.046	Wilcoxon signed rank test
JBS^c^–emotional exhaustion	3.5 (0.8)	3.7 (0.9)	.04	Wilcoxon signed rank test
JBS-depersonalization	2.1 (0.7)	2.4 (0.9)	.001	Wilcoxon signed rank test
JBS–personal achievement	3.7 (0.7)	3.7 (0.8)	.89	Wilcoxon signed rank test
STAI^d^-state	45.96 (10.6)	46.0 (12.0)	.96	2-tailed paired *t* test
STAI-trait	50.0 (9.7)	48.4 (10.4)	.09	2-tailed paired *t* test

^a^PSQI: Pittsburgh Sleep Quality Index.

^b^GHQ: General Health Questionnaire.

^c^JBS, Japan Burnout Score.

^d^STAI: State-Trait Anxiety Inventory.

Compared with the baseline, an increase in sleep duration was observed from the first week of the intervention.

**Figure 4:**
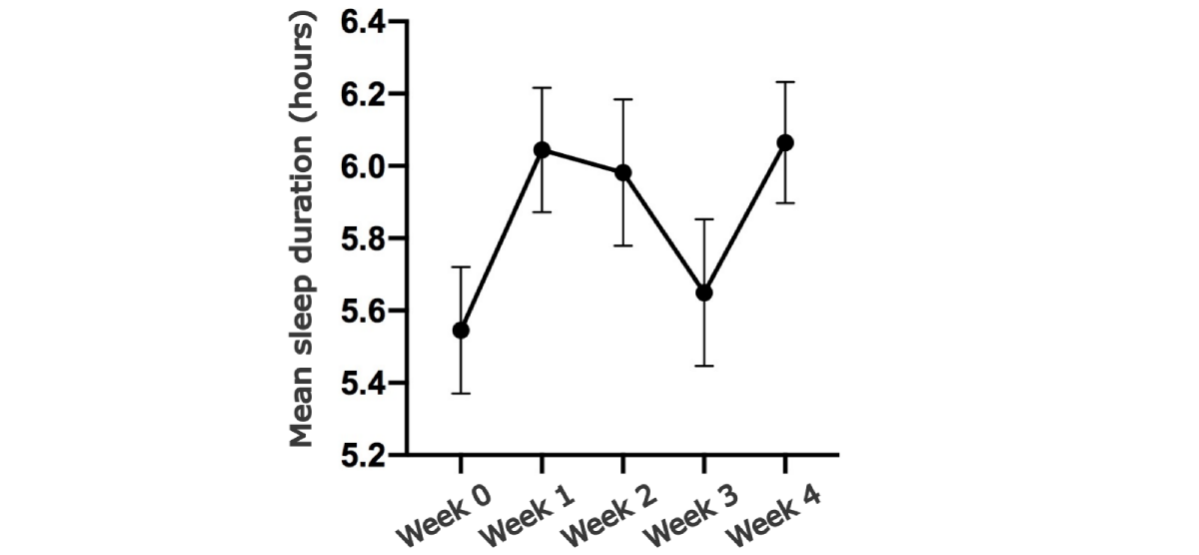
Mean sleep duration of week 0 (baseline) to week 4.

However, a decline was observed in the third week of the intervention, followed by further improvement in the fourth week (Figure 4). The cause of the observed decline during the third week remains uncertain. One plausible explanation could be attributed to the goal gradient effect, wherein participants may have exhibited heightened mindfulness toward their behavior during the final week of the study. However, it was difficult to conclude that because the app did not provide countdowns or reminders during the final week of the study. To determine the factors that contributed to the change in sleep hours from baseline to postintervention, we performed a correlation analysis. Our analysis did not reveal any correlation between the change in sleep and pretest questionnaires, age, or well-being scores (Multimedia Appendix 3). However, subgroup analysis divided by sex showed a greater improvement in female shift workers than in male shift workers. In female shift workers, the mean sleep duration increased from 5.61 hours to 6.17 hours after the intervention, whereas in male shift workers, the mean sleep duration increased from 5.72 hours to 5.84 hours (Multimedia Appendix 4). Although more participants were female, this result is consistent with that of prior studies concerning eHealth interventions [51]. However, these differences were not strong enough to have an effect on the overall change in sleep duration, shown by 3-way ANOVA including age and sex.

### Changes in Well-Being and Mental Health

There was no significant improvement in all 5 well-being scores (alertness, happiness, energy, health, and relaxation; all *P*>.05), except for an improvement of “energy” in the morning (*P*=.01). Other mental health–related questionnaires showed a significant improvement in GHQ scores (*P*=.046) and an increase in burnout scores (emotional exhaustion, *P*=.04; depersonalization, *P*=.001; Table 3).

### Postanalysis of Well-Being Prediction

After the study, the performance of the prediction model was evaluated. Postanalysis showed that the macroaveraged *F*_1_-scores for alertness, happiness, energy, health, and stress were 0.63, 0.77, 0.74, 0.77, and 0.77, respectively. In addition, a feature importance analysis was performed using random forest for all 45 variables in the prediction model. The results showed that in the three-class model, sleep duration had the value of highest importance, followed by sleep efficiency, average heart rate, and steps.

## Discussion

### Principal Findings

The main result of this study shows that physician-assisted internet-delivered CBT program in shift workers with a high risk of sleep disorders increased total sleep time, as measured using commercially available fitness trackers. After a 4-week intervention period, the mean daily sleep duration for 7 days (6.06 hours) showed a statistically significant increase compared with the baseline (5.54 hours; *P*=.02). Subjective self-rating of sleep quality also showed a statistically significant improvement.

In a systematic review of interventions for shift workers, it was found that studies that improved sleep reported increases in sleep duration ranging from 0.34 to 0.99 hours in randomized controlled trials and from 0.02 hours to 1.15 hours for nonrandomized controlled trials [52]. This study shows that sleep duration increased by 0.52 hours after the intervention, where an increase of 15 minutes can be considered clinically significant. In accordance with the results of this study, previous study results have demonstrated improvement in subjective sleep parameters after home-based or web-based interventions in medical shift workers [18,20]. This study is significant in that it shows improvement in both objective and subjective sleep parameters, further supporting the efficacy of internet-delivered CBT for sleep disorders associated with shift work.

Contrary to expectations, this study did not find an improvement in subjective well-being except for “energy” in the morning. In addition, we did not find any improvement in burnout levels. However, the feature importance analysis of well-being prediction showed that sleep duration, steps, and type of shift were factors with high relevance. A possible explanation for these contradicting results might be that sleep is simply one component of well-being or that sleep deprivation may be the result of burnout. In a study on physicians, career satisfaction, work hours, and family support were among the most relevant factors affecting well-being [53]. As this program is focused on improving sleep, the results may indicate that it is difficult to improve subjective well-being or burnout levels merely through sleep improvement. Another explanation may be that the study included shift workers with mild sleep-related disturbances. Compared with studies in patients with insomnia where sleep and mental health both improved after the intervention [13,24,25], sleep-related symptoms may not have been the main contributor to well-being in this study population. Although improvement in sleep did not result in improvement of burnout levels or subjective well-being, feature importance showed that among the several factors included in the well-being prediction model, sleep duration was the most important factor. Consistent with the existing literature, this study found that sleep was related to the subjective well-being of health care workers. In addition, improvement in sleep may also improve certain aspects of physical well-being. Further work is needed to evaluate the causal relationship between sleep and subjective well-being along with identifying specific aspects of subjective well-being intermediated by sleep deprivation.

Furthermore, one of the significant features of the proposed internet-delivered CBT program is that it combines the benefits of physician assistance with the positive aspects of self-help apps, such as promoting self-reflection and facilitating behavioral change. Several studies have demonstrated that internet-delivered CBT with the involvement of physicians or therapists is highly effective. A systematic review comparing internet-delivered therapy with face-to-face therapy found no significant differences in outcomes such as sleep efficiency, total sleep time, and insomnia severity. In 10 out of 15 trials included in the review, physician or therapist involvement, including weekly feedback, was integrated into the internet-delivered therapy [13]. Concurrently, self-help apps applying personal informatics through fitness trackers and smartphones are steadily increasing in number. Personal informatics is effective in promoting self-reflection, self-analysis, and self-guided behavior change through visual displays of continuous data and machine learning. However, researchers raised a concern that the user may incorrectly interpret data [26] or, in contrast, rely too much on algorithms to guide behavioral change [28]. This internet-based CBT program includes the advantages of personal informatics but at the same time has a lower risk of introducing misguided interpretation owing to the presence of professional advice. The findings reported here suggest that wearable sensors and personal informatics may enhance the effect of traditional CBT and promote behavioral change. Additional research is needed to confirm whether the addition of these features plays a role in internet-delivered CBT programs.

Another feature of this 4-week intervention is that the target was shift workers with sleep disorders ranging from mild to severe symptoms who were not previously diagnosed with sleep disorders or other mental health disorders. Although shift work is associated with several health issues, shift workers are essential to the health care system. Although in severe cases, adjustment of shift schedules may be the best solution, this is not usually feasible for shift workers with mild symptoms. From an occupational health perspective, interventions to leverage the health of the overall working population are needed other than providing special treatment or absence for those that exceed preventive measures. Internet-delivered intervention allows users to engage in the program on their own time, allowing flexibility and instant support, and can be tailored to the user’s needs [54]. In addition, shift workers rarely seek medical advice despite the high prevalence of sleep disorders among shift workers. Physician-assisted internet-delivered CBT will allow users to gain access to professional medical advice at an early stage. Studies comparing cost-effectiveness have shown that internet-delivered CBT is cheaper than face-to-face therapy because of less therapist time [55,56]. Considering the widespread popularity of smartphones and self-help apps, introducing internet-based CBT for shift workers with mild disturbances, and not limiting it to clinically significant individuals, may be a cost-effective way to improve occupational health.

### Limitations

Our study has a few important limitations. First, this study does not provide any conclusion to the participants’ motivation for attribution and continuation, as the research incentives were financial rewards. We acknowledge that motivation and participation are crucial factors that affect the outcomes of internet-based interventions. Nevertheless, as the incentive was merely for participation in the study and not based on any accomplishment or goals, the effect did not directly interfere with the outcome. Second, the study did not include a control group owing to the limited number of participants. We do not know whether monitoring with a consumer-targeted health tracker alone would improve the sleep-related outcomes. However, research implies that although sleep tracking devices can help users better understand and improve their overall sleep habits, excessive focus on sleep feedback provided by only the tracking devices may derail users from focusing on modifiable behaviors and sleep hygiene or may exacerbate anxiety about sleep [32,57]. Third, the study protocol lacked long-term follow-up. This study showed that sleep duration instantly improved in the first week of the intervention and temporarily declined in the third week of the intervention. We do not know if this was because they were simply told to improve their sleep habits or because of self-motivation. In addition, these results cast a question on how long it takes for a new behavior to stick without external stimuli. Although prior studies have confirmed the lasting effects of internet-based CBT on insomnia [24], we do not know if these effects lasted after the intervention in this study. These factors should be considered in future studies.

### Conclusions

Physician-assisted, internet-delivered CBT for shift workers with a high risk of sleep disorders showed significant improvement in sleep duration, as measured by wearable sensors, and subjective sleep quality. This study implies that sleep improvement programs using an app and wearable sensors are feasible and may play an important role in preventing shift work–related sleep disorders. However, no relationship was observed between sleep changes and subjective well-being. Further studies should focus on the causal relationship between daily well-being and sleep disorders related to shift work.

## Appendix

Multimedia Appendix 1 Description of the fixed-format sleep advice.

Multimedia Appendix 2 Results of ANOVA showing the effect of sex and age group on each subjective well-being score.

Multimedia Appendix 3 Heat map of the measured variables.

Multimedia Appendix 4 Change in the mean sleep duration before and after the intervention in female and male shift workers.

## Abbreviations

CBT: cognitive behavioral therapy
GHQ: General Health Questionnaire
ICU: intensive care unit
PSQI: Pittsburgh Sleep Quality Index
PVT: psychomotor vigilance test
STAI: State-Trait Anxiety Inventory
SWSD: shift worker sleep disorder
