# Supplementary figures and images for: Effect of an Internet–Delivered Cognitive Behavioral Therapy–Based Sleep Improvement App for Shift Workers at High Risk of Sleep Disorder: Single-Arm, Nonrandomized Trial
Source: J Med Internet Res. 2023 Aug 22;25:e45834. doi: 10.2196/45834 (PMC10481224; doi:10.2196/45834)

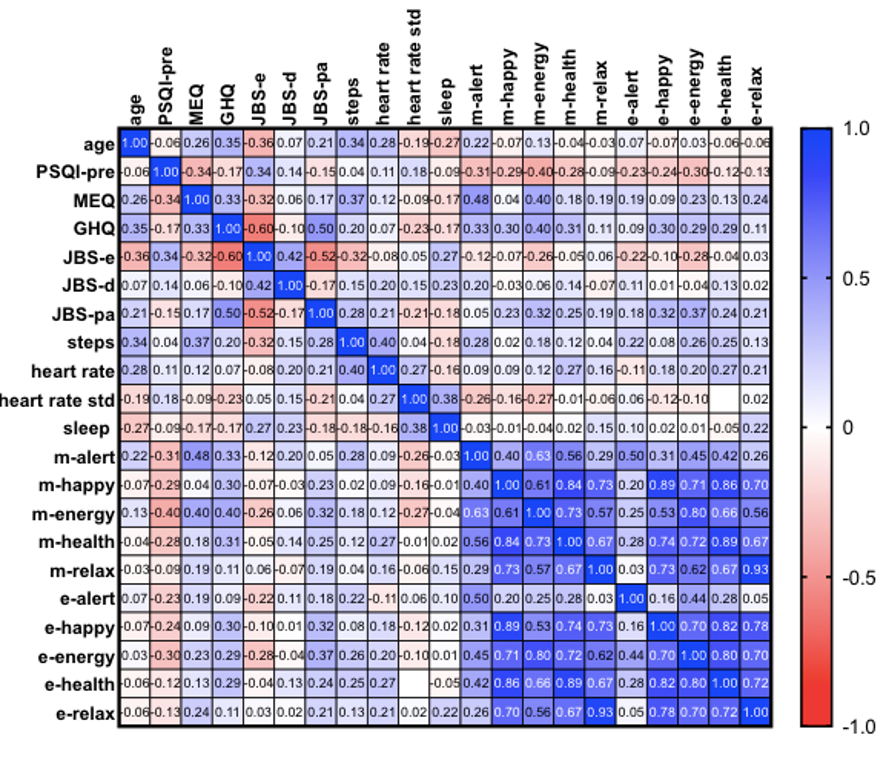

Supplement: Multimedia Appendix 3 [file jmir_v25i1e45834_app3.png]

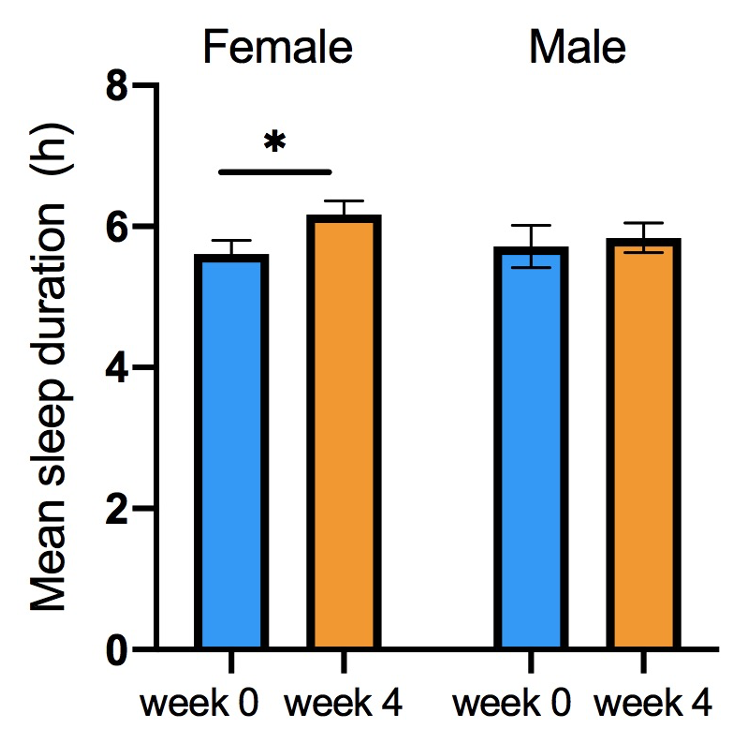

Supplement: Multimedia Appendix 4 [file jmir_v25i1e45834_app4.png]
